# Formation of Aluminum Particles with Shell Morphology during Pressureless Spark Plasma Sintering of Fe–Al Mixtures: Current-Related or Kirkendall Effect?

**DOI:** 10.3390/ma9050375

**Published:** 2016-05-14

**Authors:** Dina V. Dudina, Boris B. Bokhonov, Amiya K. Mukherjee

**Affiliations:** 1 Department of Mechanical Engineering and Technologies, Novosibirsk State Technical University, K. Marx Ave. 20, Novosibirsk 630073, Russia; 2 Lavrentyev Institute of Hydrodynamics SB RAS, Lavrentyev Ave. 15, Novosibirsk 630090, Russia; 3 Institute of Solid State Chemistry and Mechanochemistry SB RAS, Kutateladze str. 18, Novosibirsk 630128, Russia; bokhonov@solid.nsc.ru; 4 Department of Natural Sciences, Novosibirsk State University, Pirogova str. 2, Novosibirsk 630090, Russia; 5 Department of Chemical Engineering and Materials Science, University of California, Davis, 1 Shields Ave., Davis, CA 95616, USA; akmukherjee@ucdavis.edu

**Keywords:** spark plasma sintering, inter-particle, pressureless, iron aluminide, Kirkendall effect

## Abstract

A need to deeper understand the influence of electric current on the structure and properties of metallic materials consolidated by Spark Plasma Sintering (SPS) stimulates research on inter-particle interactions, bonding and necking processes in low-pressure or pressureless conditions as favoring technique-specific local effects when electric current passes through the underdeveloped inter-particle contacts. Until now, inter-particle interactions during pressureless SPS have been studied mainly for particles of the same material. In this work, we focused on the interactions between particles of dissimilar materials in mixtures of micrometer-sized Fe and Al powders forming porous compacts during pressureless SPS at 500–650 °C. Due to the chemical interaction between Al and Fe, necks of conventional shape did not form between the dissimilar particles. At the early interaction stages, the Al particles acquired shell morphology. It was shown that this morphology change was not related to the influence of electric current but was due to the Kirkendall effect in the Fe–Al system and particle rearrangement in a porous compact. No experimental evidence of melting or melt ejection during pressureless SPS of the Fe–Al mixtures or Fe and Al powders sintered separately was observed. Porous FeAl-based compacts could be obtained from Fe-40at.%Al mixtures by pressureless SPS at 650 °C.

## 1. Introduction

In electric current-assisted sintering of conductive materials, the current passes directly through the compact making inter-particle contacts parts of the electric circuit. The initial resistance of the inter-particle contacts is inherently high due to several reasons, including small diameter of the contact spot and the presence of oxide films and inter-particle gaps. Processes occurring at inter-particle contacts play a key role in the formation of bulk materials from separate powder particles and, thus, require special attention. If pulsed current is applied, the contacts that complete the electric circuit change with every pulse leading to uniform sintering [1]. The contact formation mechanisms between particles of the same material during electric current-assisted sintering have been addressed in detail in a number of studies [2,3,4,5,6,7,8,9,10,11]. Burenkov *et al.* [2,3] found evidence of electric erosion between metal particles during electric discharge sintering. Aman *et al.* [6] observed an unconventional morphology of necks formed between copper particles during pressureless Spark Plasma Sintering (SPS) and proposed an ejection mechanism of the inter-particle interactions. Modeling has shown that for fine metallic particles, due to fast heat conduction into the particle volume, no local melting at the contacts should be expected [8]. At the same time, the quality of contacts determines the thermal energy involved in sintering of the compact as a whole. Chaim [11] has recently pointed out that it is correct to discuss the plasma and spark effects for non-conducting materials only, as non-conducting particles can accumulate electric charge. In electrically conducting materials, inter-particle contacts experience excessive Joule heating. Furthermore, inter-particle contacts can be the sites of localized chemical reactions. Vasiliev *et al.* [5] suggested that a high strength of porous zeolite monoliths produced by SPS was due to strong inter-particle bonding established as a result of breakage and rearrangement of chemical bonds in the corresponding areas.

For practical applications of SPS, it is necessary to study the interaction between particles in real systems—multi-component powder mixtures. Certain steps have been made in this direction. Interdiffusion between Ni and Cu particles occurring in three dimensions during SPS was described by Rudinsky and Brochu [12]. Murakami *et al.* [13] studied the formation of compacts from Nb–Al, Nb–Al–W and Nb–Al–W mixtures during SPS with a goal to understand the mechanisms involved in the formation of dense sintered alloys. Kol’chinskii and Raichenko [14] obtained diffusion profiles in the contact region between particles of Ni and Cu formed during electric discharge sintering and laser treatment and found that the diffusion distances were twice as long as those predicted by theoretical calculations without considering the highly localized evolution of heat at the inter-particle contacts. The development of contacts between particles of dissimilar metals can be associated with the formation of solid solutions and intermetallic compounds. Enhanced reaction kinetics in Fe–Al [15] and Mo–Si [16] layered assemblies subjected to treatment in the SPS has been reported. Aiming at dense composite ceramics, Wu *et al.* [17] compared the microstructure uniformity of ceramic composites produced by pressure-assisted reactive SPS and HP and concluded that when compacts with close relative densities were obtained, those sintered by SPS tended to be more homogeneous and of a finer microstructure. This difference was attributed to high heating rates and short holding time in the SPS method. However, the peculiarities of the inter-particle interactions in compacts consolidated from binary mixtures of metals during pressureless SPS have not been investigated. In this work, we present the evolution of particle morphology in Fe–Al mixtures under conditions of pressureless SPS at 500–650 °C. We also make a comparison of the consolidation outcomes achieved by chemical reaction-accompanied pressureless SPS and sintering in a hot press without the application of electric current to the compact.

## 2. Materials and Methods

**Figure 1:**
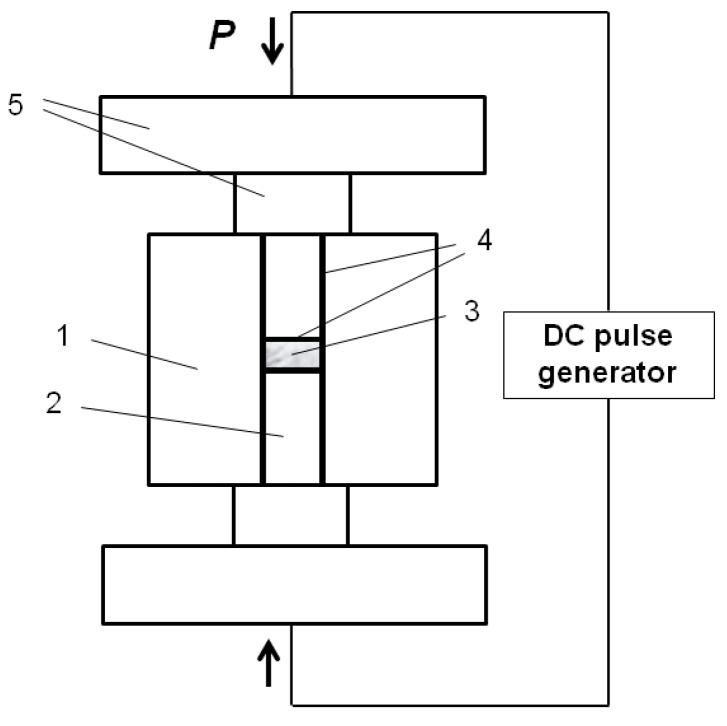
Schematic of the die/punch/spacer assembly used for pressureless Spark Plasma Sintering (SPS) experiments: (**1**) graphite die; (**2**) short graphite punches; (**3**) powder sample; (**4**) graphite foil; (**5**) graphite spacers.

For conveniently tracing the morphology changes of the particles in the sintered compacts, Fe and Al powders of spherical morphology were selected. Carbonyl iron (99%, 2.5–5 µm, “SyntezPKZh”, Dzerzhinsk, Russia) and gas-atomized aluminum (99.9%, PAD-6, average size 6 µm, “VALKOM-PM”, Volgograd, Russia) were used to prepare the Fe-40at.%Al mixtures. Spark Plasma Sintering was carried out using a SPS Labox 1575 apparatus (SINTER LAND, Inc., Nagaoka, Japan). A graphite die of a 10-mm inner diameter and 50-mm outer diameter and short graphite punches of 10 mm diameter were used. A schematic of the assembly used for the pressureless SPS experiments is shown in Figure 1. The die wall was lined with a graphite foil. Circles of graphite foil were placed between the punch and the sample. The temperature during the SPS was controlled by a К-type thermocouple NSF600 (CHINO, Tokyo, Japan) placed in the die wall at a depth of 5 mm. The maximum SPS-temperatures were 500, 600, and 650 °C. The sample was held at the maximum temperature for 3 min and then was cooled down to room temperature. Hot pressing (HP) experiments were conducted with and without applied pressure at 650 °C with a holding time of 5 min. Heating of the sample in the hot press was realized by using external heaters. The applied pressure during HP was 3 MPa. An additional 2 min of holding time were added in HP to ensure the uniform heating of the die. Both SPS and HP were conducted in vacuum. The temperature during HP was controlled by a pyrometer focused on the die wall. The heating rate was 50 °C∙min^−1^ in all SPS and HP experiments. Graphite foil was used in HP experiments in a way similar to the SPS experiments. Loose packing of the Fe-40at.%Al powder mixture corresponding to a density of 2 g∙cm^−3^ and a relative density of 38% was the initial state of the samples before consolidation, if not stated otherwise. The powder mixture was poured into SPS or HP graphite dies without any additional pressing step. A denser packing with a relative density of 65% was also used in several SPS experiments, which is specified in the specimens’ descriptions. Pure Al and pure Fe compacts were obtained starting from loose packing of the corresponding powders. During pressureless SPS and pressureless sintering in the hot press, the only load that the samples experienced was caused by the weight of the upper punch. In pressureless SPS, the die supported a certain pressure applied for electrical contact between the spacers to be established. Annealing of the powder mixture in a tube furnace was conducted at 600 °C for 30 min in a flow of argon.

The X-ray diffraction (XRD) patterns were recorded using a D8 ADVANCE diffractometer (Bruker AXS, Karlsruhe, Germany) with Cu Kα radiation. The quantitative phase analysis was conducted using Rietveld analysis of the XRD patterns in PowderCell 2.4 software [18]. The microstructure of the compacts was studied by Scanning Electron Microscopy (SEM) using a Hitachi-Tabletop TM-1000 and a Hitachi-3400S microscope (Hitachi, Tokyo, Japan). The latter is equipped with an Energy-Dispersive Spectroscopy (EDS) unit (NORAN Spectral System 7, Thermo Fisher Scientific Inc., Waltham, MA, USA). Secondary and back-scattered electron ((SE) and (BSE)) images were taken. Selective dissolution treatment of the sintered materials was conducted using 20% NaOH solution at room temperature. The open porosity of the sintered compacts was determined by filling pores with ethanol.

## 3. Results and Discussion

**Figure 2:**
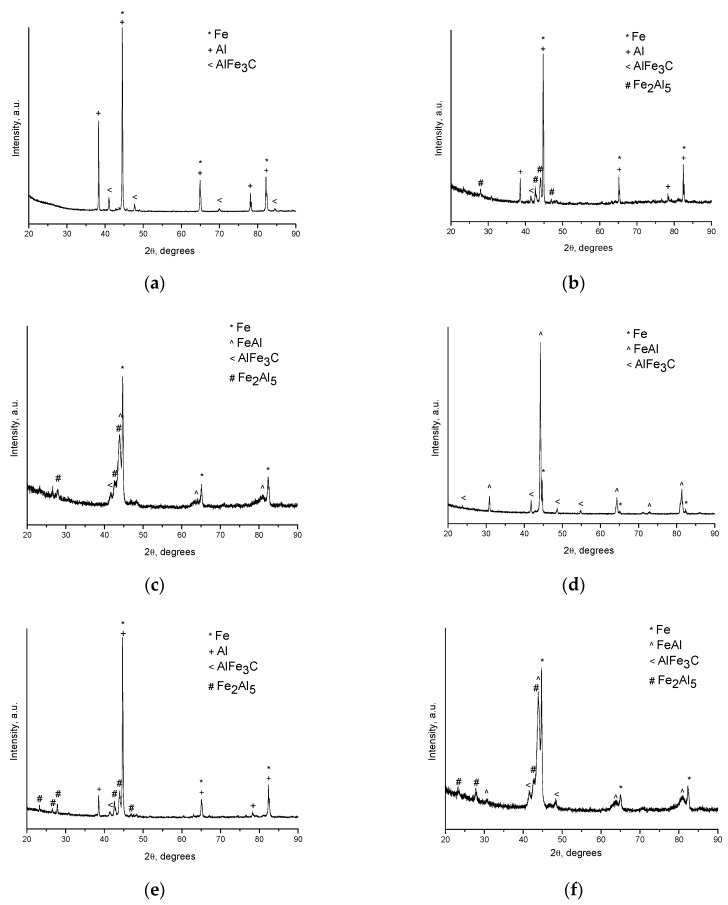
XRD patterns of the porous compacts obtained from Fe-40at.%Al mixtures by pressureless SPS at (**a**) 500 °C (green density 65%); (**b**) 600 °C; (**c**) 600 °C (green density 65%); (**d**) 650 °C and by the hot pressing technique at (**e**) 650 °C, pressureless experiment; (**f**) 650 °C, applied pressure 3 MPa.

The ternary carbide AlFe_3_C was the first phase to form at the inter-particle contacts in the porous compacts. The reflections of AlFe_3_C can be seen in the XRD pattern of the compact sintered at 500 °C (Figure 2a). In compacts sintered at higher temperatures (Figure 2b–f), the AlFe_3_C phase was also present as a minor phase. In the absence of carbon, Fe_2_Al_5_ was reported to form first upon heating of Fe–Al mixtures [19,20]. There existed a possibility of carbon diffusing from the graphite foil, similar to our previous work, in which Ni_2_W_4_C formed during SPS of Ni–W powders [21]. However, in the present study, the main source of carbon was the carbonyl iron powder itself, as the product of annealing of the Fe-40at.%Al mixtures in a flow of argon (with no external carbon sources introduced) also contained AlFe_3_C as a minor phase. The formation of AlFe_3_C in the products of reaction between a carbonyl iron powder and an aluminum powder during vacuum annealing was also reported in ref. [22]. The presence of the ternary carbide AlFe_3_C did not alter the phase sequence with increasing temperature reported for the Fe–Al system in the literature. Moreover, unexpectedly, we gained a means to show that surface layers of contacting Fe and Al particles already chemically interact during SPS at 500 °C, although this interaction is not accompanied by any noticeable morphological changes.

As was pointed out by Japka [23], the skin layer of a carbonyl iron particle etches differently compared with the rest of the particle, which is an indirect evidence of structural and chemical differences between the skin layer and the particle volume. Indeed, considering the production process of carbonyl iron powders, the concentration of carbon in the surface layer of particles can be higher than the volume-averaged value.

**Figure 3:**
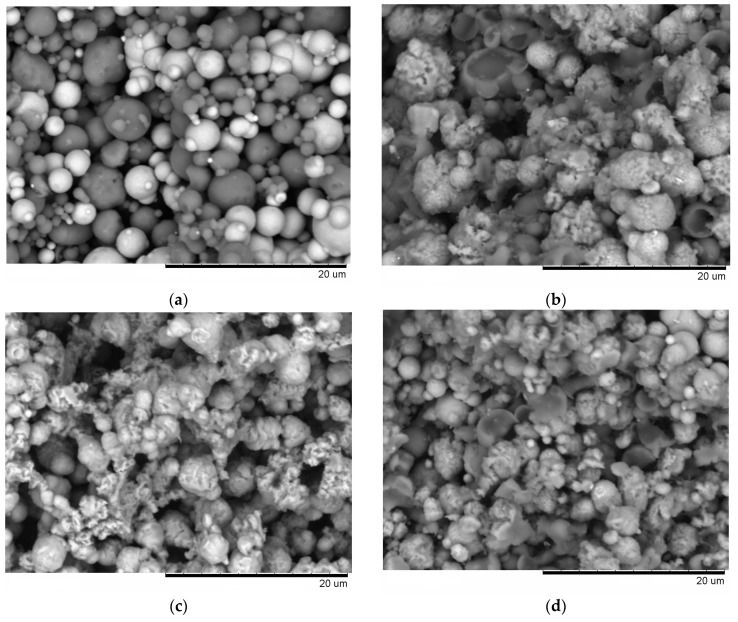
Fracture surface of porous compacts (BSE images) obtained from Fe-40at.%Al mixtures by pressureless SPS (**a**) 500 °C (green density 65%); (**b**) 600 °C; (**c**) 650 °C; (**d**) sintered in a pressureless experiment in the hot press at 650 °C.

**Figure 4:**
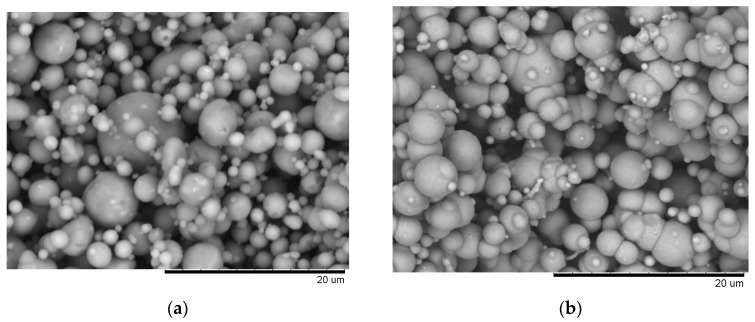
Fracture surface of porous aluminum (**a**) and porous iron (**b**) obtained by pressureless SPS at 600 °C.

From the fracture surface of the porous compacts, we can trace the evolution of the inter-particle contacts with temperature and observe the influence of green density and consolidation method of the powder (Figure 3). The spherical morphology of iron and aluminum particles in the compact sintered by SPS at 500 °C starting from a green density of 65% (Figure 3a) is largely maintained. Indeed, intense reflections of the initial components—Al and Fe—can be seen on the corresponding XRD pattern (Figure 2a). SEM did not reveal any evidence of local melting or erosion/melt ejection processes during SPS (Figure 3a). The preserved particle morphology in compacts sintered from loosely packed powders of Al (Figure 4a) and Fe (Figure 4b) powders separately at a temperature of 600 °C confirmed the absence of local melting effects, although both factors—loose initial packing and a higher temperature―could have favored non-conventional inter-particle interactions under applied current. These observations agree with modeling results of ref. [8], which showed that metallic particles several micrometers in diameter cannot sustain the locality of overheating in the inter-particle regions because of high thermal conductivity. In compacts sintered at 600 and 650 °C, because of reaction advancement, it was not possible to define the neck regions in the reaction-sintered porous compacts (Figure 3b–d), as it is usually done in compacts obtained from single-phase powders. The Fe_2_Al_5_ phase formed in the compacts processed by SPS at 600 °C starting from loose packing, although the initial reactants were still present (Figure 2b). A higher green density of the Fe-40at.%Al mixture resulted in higher transformation degrees of the reactants at the same sintering temperature (Figure 2c). This should be attributed to an increased number of the reaction initiation sites.

**Figure 5:**
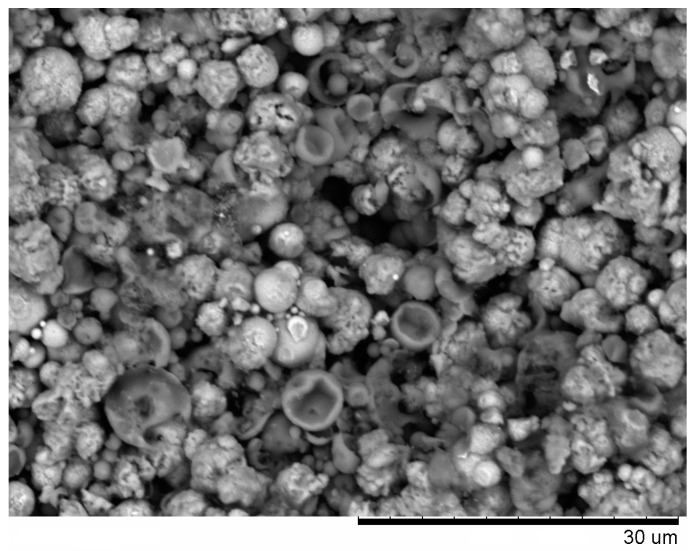
BSE image of the flat end of the disk-shaped compact Spark Plasma Sintered at 600 °C under pressureless conditions from a loosely packed Fe-40at.%Al mixture.

**Figure 6:**
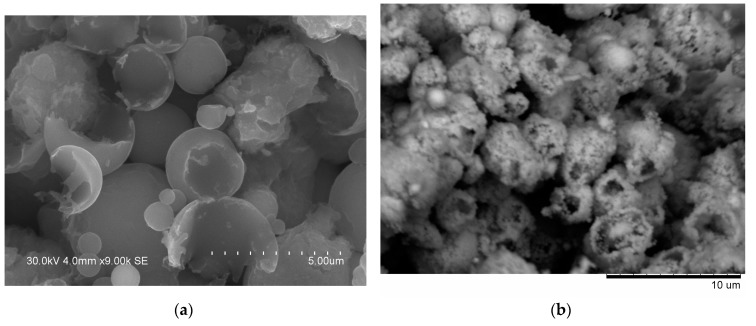
Morphology of the Al shells observed in the compacts formed by Fe-40at.%Al mixtures at an early stage of chemical interaction (**a**) and microstructure of these compacts after treatment in 20% NaOH solution (**b**); (**a**) SE image; (**b**) BSE image.

**Figure 7:**
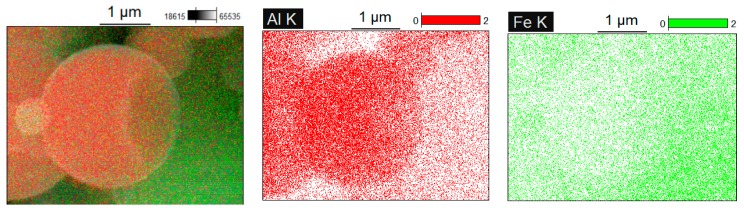
EDS mapping of particles with shell morphology observed in the compacts formed by Fe-40at.%Al mixtures at an early stage of chemical interaction.

An interesting observation made in the present study was the formation of particles with shell morphology in the compacts produced by SPS experiencing early chemical interaction stages (Figure 2b and Figure 3b). The observed morphology of seemingly “broken” shells was not due to fracturing of intact hollow particles (that could have been present in the as-sintered sample) during the preparation of samples for SEM observations, as edges of the shells showed a variety of orientations relative to the fracture surface. These shells did not show any specific orientation relative to the current direction during SPS and were also observed on the flat ends of the disk-shaped compacts (Figure 5). The flat ends of the compact were totally free from the graphite foil residue (no sticking occurred) and, therefore, did not require any manipulations to prepare a SEM sample. In a study by Rufino *et al.* [24], a fraction of the initially spherical Al particles showed cavities after heating in argon up to 700 °C, and a reasonable explanation for that was shrinkage upon solidification of the aluminum melt. In those experiments, the cavities had quite smooth edges unlike those of shells formed in the present study (Figure 6a). The EDS mapping (Figure 7) confirms that these shells are partially reacted Al particles. Some Al particles observed on the fracture surface and flat ends of the compacts are of a shape of an apple bitten from different sides. It should be noted that mechanical integrity of the contacts between particles is not maintained in the compacts sintered without the application of pressure from loose packing.

It was rather intriguing to look into the origin of the shell morphology. As comparative HP experiments have shown, particles with shell morphology also formed in the compacts consolidated without electric current (Figure 3d). The similarity of the compacts obtained by SPS and HP and showing particles with shell morphology was the early interaction stage with free aluminum still present (Figure 2b,e). As was reviewed by Anderson and Tracy [24], the synthesis of hollow particles and porous materials based on the Kirkendall effect has been conducted in a variety of systems. In the Fe–Al system, the Kirkendall pores form in places of Al particles, as Al rapidly diffuses into Fe and participates in the formation of intermetallic phases. Therefore, it was concluded that the shape of the Al particles observed in this study was due to preferential diffusion of Al into Fe and a further loss in mechanical integrity of the contact between the Al and Fe particles. As Al shells were found in the compacts produced by both SPS and HP, this morphology change was not caused by specific electric current-related effects.

Based on the data presented in Reference [25] on the thickness of the product layers grown at the interface between Fe and Al plates during SPS at 600 °C and a pressure of 5 MPa, we calculated the thickness of the Al layer consumed in the reaction in these conditions. For a holding time at the maximum temperature during SPS of 3 min, the thickness of the Al layer consumed in a planar configuration of the interface is 13 µm. Considering the diameter of the Al particles used in the present work, it may appear that the particles should have been fully consumed. However, in the configuration of Reference [25], the diffusion flow occurred in a single direction normal to the interface between the plates. In the present work, Al diffused into contacting Fe particles in several directions and in three dimensions. Furthermore, a loss in mechanical integrity of the inter-particle contacts can disrupt the diffusion flows. Treatment in NaOH solution allowed revealing another possible contact evolution scenario. The cores of the hollow particles with Fe_2_Al_5_ shells (Figure 6b) were the unreacted Al and, thus, easily dissolved in alkaline solution. This morphology was possible to form when an Al particle touched several Fe particles in the compact.

The FeAl was the major phase after SPS at 650 °C (Figure 2d), while the reaction has only started by forming a small quantity of Fe_2_Al_5_ in the compact processed by pressureless HP at this (measured) temperature (Figure 2e). Even applying a pressure during HP did not result in the same transformation degree as was achieved during SPS (Figure 2d,f). Passing electric current through a mixture of powders is used to initiate a combustion reaction for the synthesis of the target products, if the reaction mixture is conductive [26]. In porous compacts, the inter-particle contacts have to carry high current densities [27], which enhance the diffusion kinetics at the interfaces in the case of dissimilar contacts-contacts between the reactants. As the calculated content of free iron in the compact Spark Plasma Sintered at 650 °C from the Fe-40at.%Al mixtures was only 5 vol.%, it can be concluded that reactive SPS offers a very fast synthesis route of porous FeAl-based materials. The open porosity in this compact (Figure 3c) was 42% of the total compact volume.

From a technological perspective, this work has shown that pressureless reactive SPS is a fast synthesis method of porous Fe–Al intermetallics, which are promising high-temperature materials for environmental applications, such as filtration of gases and liquids containing corrosive species. In our experiments, we have also attempted reactive sintering of the Fe–Al mixtures using a SPS die/punch configuration without the upper punch. We found that the absence of direct contact between the compact and the punch causes significant gradients in the Fe–Al compacts, seen both in the phase composition and microstructure. Therefore, in order to ensure the uniformity of the phase composition, microstructure and pore structure of the FeAl porous intermetallic sintered by SPS, direct contacts between the compact and the two punches should be maintained during sintering.

## 4. Conclusions

The features of interaction between particles of Fe and Al having diameters of several micrometers forming a porous compact during pressureless SPS were studied. The phase evolution of the system with temperature was traced. At early interaction stages, Al particles acquired shell morphology. It was confirmed that the formation of shells was not related to the influence of electric current but was due to the Kirkendall effect in the Fe–Al system and particle rearrangement in a porous compact. No experimental evidence of local melting or erosion/melt ejection processes during SPS was found.

This study has shown that inter-particle interactions between particles of dissimilar materials are more complex than interactions between particles of the same material during SPS in terms of morphology evolution and morphological changes observed during SPS of reacting systems should be carefully studied to separate the effects related to chemical interaction from those caused by passing current, if any.

## Abbreviations

The following abbreviations are used in this manuscript:

SPS	Spark Plasma Sintering
HP	Hot Pressing
XRD	X-ray diffraction
SEM	Scanning Electron Microscopy
SE	Secondary Electron
BSE	Back-Scattered Electron
EDS	Energy-Dispersive Spectroscopy
